# *APOE* ε4-related differences in brain structure, function, and connectivity at midlife: A scoping review

**DOI:** 10.1016/j.tjpad.2025.100364

**Published:** 2025-08-29

**Authors:** Rikki Lissaman, Sidra Anjum, Andrea Quaiattini, M. Natasha Rajah

**Affiliations:** a Department of Psychiatry, McGill University, Montreal, QC, Canada; b Department of Psychology, Royal Holloway, University of London, Egham, Surrey, UK; c Centre for Addiction and Mental Health, Toronto, ON, Canada; d Schulich Library of Physical Sciences, Life Sciences, and Engineering, McGill University, QC, Canada; e Department of Psychology, Toronto Metropolitan University, Toronto, ON, Canada

**Keywords:** Apolipoprotein E, Midlife, Brain structure, Brain function, Structural connectivity, Functional connectivity

## Abstract

**Background:**

The apolipoprotein E (*APOE*) ε4 allele is a major genetic risk factor for late-onset Alzheimer’s disease (AD), yet there is little consensus about how and when the allele exerts its influence on the brain.

**Methods:**

In this scoping review, we synthesized research examining *APOE* ε4-related differences on MRI-derived measures of brain structure, function, and connectivity in cognitively unimpaired, middle-aged adults (aged 40–65 years). Four online databases (Ovid MEDLINE, Ovid Embase, Ovid PsycINFO, Scopus) were searched on July 11, 2024, and forward/backward reference searching was conducted on identified studies. We extracted data on sample characteristics, methods, and key *APOE* ε4-related results.

**Results:**

Our pre-registered search strategy identified 30 relevant studies. Overall, we found little evidence of robust, consistent differences between *APOE* ε4 carriers and non-carriers at midlife, especially in relation to brain structure. However, among the studies identified, small samples were common, and limited consideration was afforded to factors such as sex and ethnocultural diversity.

**Conclusion:**

Overall, the existing literature indicates that *APOE* ε4 exerts little, if any, influence on brain structure at midlife, while differences in brain function and connectivity remain poorly characterized.

## Highlights

• We review *APOE* ε4-related differences in the middle-aged brain.
• Current research provides little evidence of robust differences.
• Small, non-representative samples were prominent.
• Few studies considered the role of sex-specific factors or ethnocultural diversity.

## Graphical abstract

**Figure:**
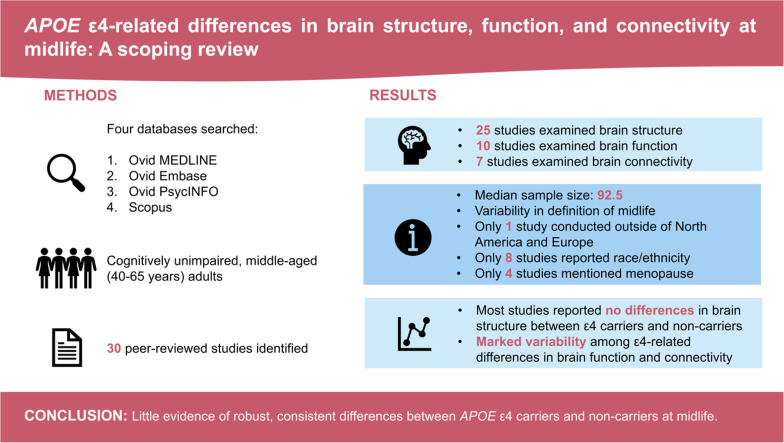

## 1 Introduction

The human apolipoprotein E (*APOE*) gene, located on chromosome 19, encodes a multi-functional protein, APOE, originally identified for its role in lipid metabolism [1,2]. There are three common allelic variants of the *APOE* gene: ε2, ε3, and ε4. The APOE protein isoforms produced by these three alleles differ in their amino acid sequences at only two positions [3,4]. Although small, these single amino acid differences alter the structure and function of APOE [2], [5] and, in turn, have significant implications for an individual’s risk of developing late-onset Alzheimer’s disease (AD; [6]). Relative to the most common *APOE* genotype (ε3/ε3), possession of the ε4 allele is associated with a ∼2- to 4-fold and ∼8- to-12-fold increase in AD risk among heterozygote and homozygotes, respectively [7,8]. Estimates of lifetime risk for AD, as opposed to odds ratios, indicate that the risk conferred by *APOE* ε4 is closer in magnitude to major genes in Mendelian diseases than to genetic loci identified through genome-wide association studies [9]. *APOE* ε4 is also strongly associated with earlier and more advanced accumulation of amyloid-β [[10], [11], [12]], a cardinal pathological feature of AD [13]. Accordingly, although not all *APOE* ε4 carriers ultimately develop AD, the allele is widely recognized as a major genetic risk factor for late-onset AD.

Given the *APOE* ε4-AD link, there is considerable research interest in understanding how and when the allele exerts its influence on the brain. To this end, magnetic resonance imaging (MRI) – a non-invasive imaging modality capable of evaluating brain structure, function, and connectivity in vivo – has been widely adopted. To date, however, results have been mixed. Among older adults, *APOE* ε4 has been associated with lower gray matter volume in regions implicated in AD, most notably the hippocampus, although findings are not consistent (e.g., [[14], [15], [16]]; for reviews, see [17,18]). Studies using diffusion MRI to probe *APOE* ε4-related differences in the microstructural properties of white matter – the brain’s structural connections – have likewise produced heterogenous findings, both in terms of the presence/absence of differences and the tracts implicated (e.g., [15], [19], [20], [21], [22]). In terms of brain function, task-evoked functional MRI (fMRI) studies utilising episodic memory paradigms have reported higher and lower activation levels in older ε4 carriers compared to non-carriers, as well as null effects [23]. To account for these inconsistencies, some researchers have turned to the prodromal hypothesis [24,25], which posits that ε4-related differences in brain health and cognition emerge late in life (∼65+ years), fueled by greater levels of AD pathology (e.g., amyloid-β) among ε4 carriers who will ultimately develop AD [26]. According to this perspective, inconsistencies between studies may be driven by the incidental inclusion (or exclusion) of older ε4 carriers in the prodromal phase of AD.

However, *APOE* ε4-related differences in brain structure, function, and connectivity have been observed much earlier in the adult lifespan, decades prior to the onset of significant levels of AD pathology. For instance, several studies have reported that young adult ε4 carriers compared to non-carriers exhibit higher levels of functional activation and connectivity, especially in AD-vulnerable regions such as the hippocampus and posteromedial cortex [27], [28], [29], [30], [31], [32]. Recent work has further reported an association between heightened memory-related activation in posteromedial cortex and subsequent amyloid-β burden in older ε4 carriers [33]. Such findings have led some to propose lifespan accounts, whereby *APOE* ε4 induces a prolonged pattern of “hyperactivation”, maintained throughout early-/mid-adulthood, predisposing specific brain networks to the accumulation of AD pathology [34,35]. Other researchers, however, have argued that neural differences in early adulthood may be independent of *APOE* ε4’s role in AD [36], [37], [38]. Proponents of the antagonistic pleiotropy hypothesis [39,40], for example, argue that possession of the ε4 allele is evolutionary advantageous in early life but detrimental in later life. In this context, heightened levels of activation among young adult ε4 carriers are considered beneficial, unrelated to later AD risk [26]. It is currently unclear whether these benefits are maintained into midlife, as some have suggested (e.g., [41]), or whether midlife represents a transition period characterized by minimal ε4-related differences as the allele’s influence shifts from advantageous to disadvantageous [42].

Despite this, little is currently known about *APOE* ε4-related differences in the brain at midlife. Although historically understudied, midlife – broadly defined as ages 40 to 65 years – is increasingly recognized as a pivotal point in the adult lifespan, shaping both normal and pathological brain health trajectories [43], [44]. Many factors, both biological (e.g., menopause) and lifestyle (e.g., physical exercise, diet), contribute to the importance of this period and its impact on the brain and cognition [45], [46], [47]. Nevertheless, it remains to be seen whether midlife also marks a transition point for the influence of *APOE* ε4. In terms of cognition, findings to date have been mixed, providing little evidence of a well-defined cognitive profile associated with *APOE* ε4 at midlife [48]. However, this does not preclude the presence of neural differences. For instance, lifespan accounts specifically argue that while *APOE* ε4-related “hyperactivation” is present throughout midlife, it is a precursor to later life cognitive decline [34,35]. To assess such possibilities and inform our understanding of when and how *APOE* ε4 influences the brain, a comprehensive review of the extant literature is warranted. To our knowledge, only one review has specifically targeted midlife [49], yet the authors did not incorporate a lower age cut-off, leading to the inclusion of many studies containing younger adults, complicating interpretation.

In this scoping review, we attempt to address this knowledge gap. Our primary aim was to systematically summarize what is currently known about *APOE* ε4-related differences on MRI-derived measures of brain structure, function, and connectivity in cognitively unimpaired, middle-aged adults (aged 40–65 years). Our secondary aim was to summarize the methods used to date, highlighting potential issues and outstanding questions for future research.

## 2 Methods

We used the Preferred Reporting Items for Systematic Reviews and Meta-Analyses extension for Scoping Reviews (PRISMA-ScR) checklist [50] to ensure this scoping review was transparently and clearly described. Ethical approval was not required.

### 2.1 Protocol and registration

Our protocol was developed a priori and pre-registered on the Open Science Framework (https://doi.org/10.17605/OSF.IO/5S4K9). No substantive deviations were made. However, during screening, we encountered studies that led us to add additional details, clarifying our approach. All such clarifications are provided in the relevant sections below.

### 2.2 Information sources and search strategy

The search strategy was developed for Ovid MEDLINE by a subject librarian (A.Q.) working alongside a member of the review team (R.L.). Once finalized, the search strategy was translated to Ovid Embase, Ovid PsycINFO, and Scopus. A combination of text-word and controlled vocabulary terms were used to target key concepts of interest: *APOE* and MRI-derived measures of brain structure, function, and connectivity. To maximize inclusivity, search terms related to midlife were not included. Instead, we screened studies for relevance. To limit the number of publications requiring screening, however, we included a term to remove animal only studies, where possible.

All searches were run on July 11, 2024. No language limits were applied. The full search strategy is provided in the Supplementary Material. For completeness, we also performed forward and backward reference searching of studies that passed full-text review. Forward reference searching was performed using Scopus and restricted to studies published on or before July 11, 2024.

### 2.3 Eligibility criteria

All studies identified in our searches were assessed against multi-stage eligibility criteria. First, studies had to be published in a peer-reviewed journal. Owing to the language proficiency of the authors, studies also had to be written in English. Second, studies had to include a distinct group of cognitively unimpaired, middle-aged adults. For this review, we define “cognitively unimpaired” as the absence of participants with neurological/psychiatric disorders and “middle-aged adults” as those aged between 40 and 65 years [43]. To meet this age criterion, studies had to explicitly state the age range of the included participants or the age range used for (initial) recruitment. In addition, if studies included more than one age group, they had to include an *APOE* ε4 comparison within the middle-aged group. Third, studies had to use MRI to examine brain structure, function, or connectivity. For this review, “structure” refers to volumetric and/or cortical thickness measures derived from structural MRI, whereas “function” refers to task-evoked BOLD signal change (i.e., activation/deactivation), assessed using fMRI. We use the term “connectivity” to refer to the temporal synchronicity of the BOLD fMRI signal across regions during task or rest (i.e., task-based or resting-state functional connectivity) and diffusion MRI-derived measures assessing the microstructural properties of white matter (i.e., structural connectivity). We thus excluded studies using MRI methods that do not assess these neural properties, as well as non-MRI methods. This ensured that we covered studies examining the neural properties of interest at a broadly similar spatial and temporal scale. Fourth, studies had to directly compare a group of *APOE* ε4 carriers to one or more groups of non-carriers. We did not include studies that adopted genome-wide association-like analyses, whereby associations with many single nucleotide polymorphisms in/near the *APOE* locus were examined. Moreover, if studies incorporated other factors (e.g., family history of dementia), we included them only if one of two conditions were met: (1) a formal statistical comparison between a carrier and non-carrier group at the same level of the other variable was presented (e.g., *APOE* ε4 carriers with a positive family history of dementia vs. *APOE* ε4 non-carriers with a positive family history of dementia); (2) a “main effect” of *APOE* ε4 was reported from the statistical model. It was not enough to adjust for *APOE* ε4 carrier status.

### 2.4 Screening and data extraction

Our search results were uploaded into Covidence (Veritas Health Innovation, Melbourne, Australia) for title and abstract screening. This screening step was performed independently by two reviewers (R.L., S.A.) who adopted a liberal approach, excluding studies only if there was evidence that they did not meet our stated eligibility criteria. For example, if a study abstract did not include an age range, we initially included it (if other criteria were satisfied). Disagreements were discussed and, where necessary, submitted to a third reviewer (M.N.R.) for mediation. All studies that passed this initial step were then subject to full-text review, again completed independently by two reviewers (R.L., S.A.) and mediated by a third (M.N.R). This step was performed more conservatively. For example, if a study passed title and abstract screening due to lack of evidence (e.g., no age range in the abstract), it was excluded during full-text review if the age range was either (a) not specified in the methods or (b) incompatible with our definition of midlife.

Data from studies that passed full-text review were extracted by one reviewer (R.L.) using a project-specific Microsoft Excel spreadsheet. The extracted data included general publication information (e.g., title, publication year), methods (e.g., cross-sectional/longitudinal design, imaging modalities used), sample characteristics (e.g., sample size), and key *APOE* ε4-related results.

### 2.5 Synthesis of results

Studies were grouped according to whether they assessed brain structure, function, or connectivity. We initially planned to include separate structural and functional connectivity sections. However, due to the small number of studies using diffusion MRI, we instead grouped these studies together. Note that studies adopting a multi-modal approach were included in more than one category. Moreover, given the variable statistical methods and corresponding *p*-value thresholds used across studies, we discuss results according to the methods used in each study, as done elsewhere [51].

## 3 Results

### 3.1 Search results

**Fig. 1:**
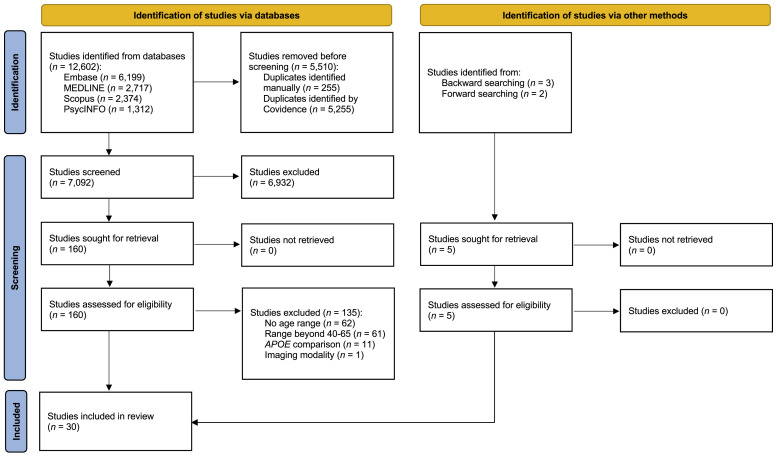
PRISMA flow diagram for the scoping review.

A PRISMA flow diagram outlining our search results is provided in Fig. 1. After removing duplicates, our initial searches yielded 7092 studies. Of these, 6932 were excluded during title and abstract screening, leaving 160 studies for full-text review. In total, 135 studies were subsequently excluded. Reasons for exclusion were: age range not reported (*n* = 62); age range did not fall between 40 and 65 years (*n* = 61); lack of relevant *APOE* ε4 comparison (*n* = 11); did not use neuroimaging modality of interest (*n* = 1). Given the large number of studies excluded for failing to report an age range, we conducted a follow-up analysis to estimate the age range for these studies. As shown in the Supplementary Material, only one of the 62 studies reported values potentially consistent with a range between 40 and 65 years, suggesting our age criteria were appropriate for this midlife-specific review. The reference lists of the remaining 25 studies were then scanned, leading to the identification of three additional studies [52], [53], [54]. All three studies included relevant *APOE* ε4-related analyses, even though *APOE* was not mentioned in the title or abstract. Forward reference searching, performed in Scopus, led to the identification of two further studies [55,56]. These studies likewise did not mention *APOE* ε4 in the title or abstract. In total, therefore, we identified 30 studies eligible for this scoping review.

### 3.2 Study characteristics

Among the 30 studies identified, 25 focused on brain structure (83.3 %), 10 focused on brain function (33.3 %), and 7 focused on brain structural/functional connectivity (23.3 %). These numbers do not sum to 30 as more than a third of the included studies were multi-modal (*n* = 11, 36.7 %). Collapsed across modality, most studies (*n* = 25, 83.3 %) adopted a cross-sectional design or reported only cross-sectional analyses of *APOE* ε4. In terms of sample size, substantial variability was evident. Median total sample size across studies was 92.5 (range = 32–611). When separated according to the neural properties examined, however, we found that studies focused on structure tended to include larger samples (*Mdn* = 122) than those focused on function (*Mdn* = 52) or connectivity (*Mdn* = 46). Surprisingly, we found little evidence to indicate that sample sizes had increased over the last two decades (Supplementary Figure 1).

**Fig. 2:**
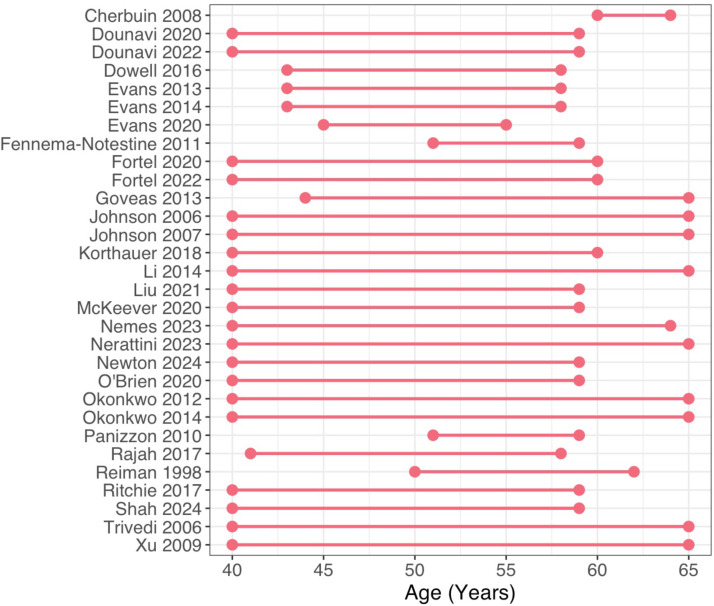
Age range in midlife *APOE* ε4 studies. Studies are listed by first author name and year of publication, arranged alphabetically. Circles represent minimum and maximum age values, while lines represent the full range. The ranges shown here reflect the full age range of the included participants or, if not available, the age range used for recruitment.

Variability was also evident in the age range of participants (Fig. 2). For instance, while all but one study [57] included individuals aged 50–55 years, those aged 40–55 and 60–65 years were less consistently included. In terms of geography, we found that most studies were conducted in North America (*n* = 17, 56.7 %) and Europe (*n* = 12, 40 %), although one study was conducted in Oceania (*n* = 1, 3.3 %). None of the studies included were conducted in Africa, Asia, or South America. Furthermore, only 8 studies provided information on the racial/ethnic composition of the included participants [56], [58], [59], [60], [61], [62], [63], [64]. The reported values demonstrate that most of the participants (≥ 75 %) identified as white or “Caucasian”.

Almost all studies included here were mixed-sex^1^ studies (*n* = 27, 90 %). Among the three single-sex studies, two focused on males [63], [65] and one focused on females [61]. Notably, excluding one study that did not report figures for sex [66], all mixed-sex studies included more females than males. It was surprising, therefore, that only four studies in this midlife review mentioned menopause. Of these, two discussed menopause in their discussion [60], [67], one reported the number of (self-reported) menopausal females in the sample but did not collect further details [68], and one (female-only) study reported the number of pre-, peri‑, and post-menopausal females included, while examining associations between gonadotropins and neuroimaging biomarkers [61]. None of the studies examined the potential link between *APOE* ε4-related differences and menopausal status, despite the skew toward females.

### 3.3 Brain structure

Table 1 provides an overview of the 25 studies that examined differences in brain structure between midlife *APOE* ε4 carriers and non-carriers. Most of these studies focused on volumetric measures, although some examined cortical thickness. Whole-brain analyses tended to use voxel-based morphometry, but others analyzed global/whole-brain measures (e.g., total grey matter volume). Region of interest (ROI) analyses were heavily focused on the hippocampus. Of the 19 studies reporting an ROI analysis, 17 (89.5 %) included the hippocampus as an ROI, while 10 (52.9 %) included the hippocampus as the only ROI.

**Table 1:** Summary of studies examining *APOE* ε4-related differences in brain structure at midlife.

Study	Sample *N*	Age range	Design	Analytical approach	Key *APOE* ε4 result(s)
Cherbuin et al. [57]	331 (89 ε4+)	60–64	Cross-sectional	Whole-brain & ROI (hippocampus, amygdala)	(1) No difference in total GM or WM volume (2) No difference in ROI volumes (3) No difference in whole-brain VBM analysis
Dounavi et al. [69]	180 (67 ε4+) / 156 (59 ε4+)	40–59	Longitudinal	ROI (hippocampus + subfields)	(1) No difference in hippocampal volume at baseline or two-year follow-up (2) Lower molecular layer volume at baseline in ε4+
Dounavi et al. [70]	600 (233 ε4+)	40–59	Cross-sectional	Whole-brain & ROI (hippocampus + subfields, assorted subcortical structures)	(1) No difference in ROI volumes (2) No difference in cortical thickness
Dowell et al. [71]	37 (17 ε4+)	43–58	Cross-sectional	ROI (parahippocampus, cuneus, anterior cingulate, precuneus, posterior cingulate, hippocampus)	(1) No difference in ROI (WM) volumes (2) Thicker parahippocampal cortex in ε4+
Evans et al. [72]	32 (16 ε4+)	45–55	Cross-sectional	ROI (hippocampus)	(1) No difference in hippocampal volume
Fennema-Notestine et al. [65]	482 (125 ε4+)	51–59	Cross-sectional	Whole-brain & ROI (assorted medial temporal, lateral temporal, and frontal regions)	(1) Thinner frontal cortices in ε4+ (2) Thicker fusiform cortex in ε4+
Goveas et al. [73]	46 (20 ε4+)	44–65	Cross-sectional	Whole-brain	(1) No difference in whole-brain VBM analysis
Johnson et al. [74]	132 (48 ε4+)	40–65	Cross-sectional	Whole-brain	(1) No difference in whole-brain VBM analysis
Johnson et al. [75]	110 (39 ε4+)	40–65	Cross-sectional	Whole-brain	(1) No difference in whole-brain VBM analysis
Li et al. [76]	46 (20 ε4+)	44–65	Cross-sectional	Whole-brain & ROI (hippocampus)	(1) No difference in whole-brain VBM analysis (2) No difference in hippocampal volume
Liu et al. [52]	160 (61 ε4+)	40–59	Longitudinal	Whole-brain	(1) No difference in total GM or WM volume at baseline or two-year follow-up (2) No difference in total cortical thickness
McKeever et al. [53]	150 (57 ε4+)	40–59	Cross-sectional	ROI (hippocampal subfields)	(1) No difference in hippocampal subfield volumes
Nemes et al. [60]	77 (33⁺ ε4+)	40–64	Cross-sectional	Whole-brain & ROI (hippocampus)	(1) No difference in whole-brain VBM analyses (males or females, respectively) (2) No difference in hippocampal volume
Nerattini et al. [61]	191 (86⁺ ε4+)	40–65	Cross-sectional	ROI (frontal regions)	(1) No difference in frontal GM volume
Newton et al. [55]	53 (18 ε4+)	40–59	Cross-sectional	ROI (entorhinal cortex + subregions, hippocampal subfields, retrosplenial cortex, posterior cingulate)	(1) No difference in ROI volumes
O'Brien et al. [54]	165 (64 ε4+)	40–59	Longitudinal	Whole-brain	(1) No difference in brain volume change
Okonkwo et al. [62]	108 (35 ε4+)	40–65	Longitudinal	Whole-brain & ROI (posterior cingulate, hippocampus, parahippocampal gyrus, amygdala)	(1) No difference in ROI-restricted VBM analysis at baseline or four-year follow-up (2) Lower right middle temporal gyrus GM density in ε4+ (exploratory whole-brain VBM analysis)
Okonkwo et al. [56]	122 (48⁺ ε4+)	40–65	Cross-sectional	ROI (hippocampus)	(1) No difference in hippocampal volume
Panizzon et al. [63]	375 (94⁺ ε4+)	51–59	Cross-sectional	ROI (hippocampus)	(1) Lower hippocampal volume in ε4+ (only when *APOE*-testosterone interaction present)
Rajah et al. [68]	49 (11 ε4+)	41–58	Cross-sectional	ROI (hippocampus)	(1) No difference in hippocampal volume
Reiman et al. [66]	33 (11 ε4+)	50–62	Cross-sectional	ROI (hippocampus)	(1) No difference in hippocampal volume (2) No difference in left-right asymmetry of hippocampal volume
Ritchie et al. [64]	208 (75⁺ ε4+)	40–59	Cross-sectional	Whole-brain & ROI (hippocampus)	(1) No difference in total brain volume of whole-brain grey matter (2) No difference in hippocampal volume
Shah et al. [77]	611 (235 ε4+)	40–59	Cross-sectional	ROI (thalamus + subregions)	(1) No difference in thalamus volume (2) No difference in thalamic subregion volumes
Trivedi et al. [78]	40 (23 ε4+)	40–65	Cross-sectional	ROI (medial temporal lobe)	(1) No difference in ROI-restricted VBM analysis
Xu et al. [79]	74 (33 ε4+)	40–65	Cross-sectional	Whole-brain	(1) No difference in whole-brain VBM analysis

*Note*. Sample *N* reflects the total number of cognitively healthy middle-aged participants reportedly included in relevant *APOE* ε4 analyses. The number of *APOE* ε4 carriers (ε4+) is provided in parentheses. Age range is reported in years and reflects the stated age range of the included participants or, if unavailable, the age range used for (initial) recruitment. We did not differentiate between studies adopting different definitions of *APOE* ε4+ (e.g., ε3/ε4 vs. ε3/ε4 + ε4/ε4). For studies with multiple groups (e.g., FH+/ε4+, FH-/ε4+, etc.), we summed across carrier/non-carrier groups. For studies reporting the percentage of *APOE* ε4 carriers, we used the reported sample sizes to calculate values and, where necessary, rounded to the nearest whole number (indicated by ⁺). Design reflects the cross-sectional or longitudinal nature of the *APOE* ε4 analysis and not necessarily the overall design of the study.

Abbreviations: *APOE* = apolipoprotein-E; FH = family history of dementia; GM = grey matter; ROI = region of interest; VBM = voxel-based morphometry; WM = white matter.

Among the 25 studies reviewed, 20 (80 %) did not observe a statistically significant difference between *APOE* ε4 carriers and non-carriers. While null findings were prominent in studies employing voxel-based morphometry, studies examining whole-brain and ROI-specific measures of volume and cortical thickness also reported null findings (Table 1). Moreover, even among the 5 studies reporting at least one significant *APOE* ε4-related difference [62], [63], [65], [69], [71], no consistent pattern was evident. For instance, in a single-sex (male-only) study, Fennema-Notestine et al. [65] reported that ε4 carriers possessed thinner frontal cortices but thicker fusiform cortices than ε4 non-carriers. However, no such differences were observed in the thickness of parahippocampal cortex, despite another study reporting ε4-related differences in this region at midlife [71]. Fennama-Notestine et al. also observed no difference in hippocampal volume, consistent with most midlife studies reviewed here, but inconsistent with a study conducted on participants from the same male-only cohort [63]. That said, the negative influence of *APOE* ε4 on hippocampal volume in the latter study was only evident when the interaction between the allele and testosterone was included in the statistical model [63].

The focus on the whole hippocampus and not its subfields may also explain the lack of ε4-related differences in studies targeting this structure. While terminology varies, it is generally accepted that the hippocampus includes distinct subfields: the cornu ammonis fields, dentate gyrus, and subiculum [80]. There is evidence to suggest that these subfields are differentially impacted in the early stages of AD [81] and thus differences between *APOE* ε4 carriers and non-carriers may likewise be present earlier in the lifespan. Accordingly, by averaging across subfields, it is possible that important differences were missed. This view is partially supported by findings from Dounavi et al. [69] who reported that the volume of the hippocampal molecular layer, but not other hippocampal subfields, was lower in ε4 carriers. However, the same group failed to replicate this result in a subsequent study adopting a different method for the automated segmentation and processing of hippocampal subfields [70]. Additionally, two other studies featured in this review – one including manual segmentation of hippocampal subfields at 3T [53], one including semi-automated segmentation of hippocampal subfields at 7T [55] – failed to identify ε4-related differences.

### 3.4 Brain function

Table 2 provides an overview of the 10 midlife studies that used fMRI to examine *APOE* ε4-related differences on brain function. Analytical approaches varied across studies: four adopted whole-brain analyses (40 %), two adopted ROI-specific analyses (20 %), and four conducted mixed analyses (40 %). As with studies of brain structure, ROI-specific analyses often focused on the hippocampus and medial temporal lobe, although other regions (e.g., posterior cingulate cortex) were also examined. Interestingly, many of these midlife fMRI studies (*n* = 7, 70 %) reported at least one significant difference between ε4 carriers and non-carriers, but variability was present.

**Table 2:** Summary of studies examining *APOE* ε4-related differences in brain function at midlife.

Study	Sample *N*	Age range	Design	Task(s)	Analytical approach	Key *APOE* ε4 result(s)
Evans et al. [67]	36 (17 ε4+)	43–58	Cross-sectional	Covert attention	Whole-brain & ROI (hippocampus, extrastriate visual areas, inferior parietal areas, anterior cingulate areas, right middle frontal areas)	(1) Lower activation in extrastriate cortex independent of cue type (valid, invalid) among ε4+
Evans et al. [82]	40 (19 ε4+)	43–58	Cross-sectional	Prospective memory, covert attention	Whole-brain & ROI (hippocampus, superior parietal areas)	(1) Lower activation in extrastriate cortex and right superior parietal lobule during prospective memory trials among ε4+ (2) No difference in activation for validity effect (valid trial mean RTs - invalid trial mean RTs) on covert attention task
Evans et al. [72]	32 (16 ε4+)	45–55	Cross-sectional	Old/new recognition	Whole-brain & ROI (hippocampus, parahippocampus)	(1) No difference in activation for subsequent memory effects (2) Greater activation in left hippocampus (subiculum) for remembered vs. forgotten items in ε4- but not ε4+
Johnson et al. [74]	132 (48 ε4+)	40–65	Cross-sectional	Old/new recognition	Whole-brain	(1) No difference in activation for novelty effect (new > old)
Johnson et al. [75]	110 (39 ε4+)	40–65	Cross-sectional	Self-appraisal	Whole-brain	(1) Greater activation for referential self-appraisal in left superior frontal gyrus, left anterior cingulate, and retrosplenial areas among ε4+ with negative FH (2) No difference in activation for referential self-appraisal (restricted to regions not showing interaction)
Newton et al. [55]	53 (18 ε4+)	40–59	Cross-sectional	Spatial memory	ROI (posteromedial entorhinal cortex)	(1) No difference in grid cell-like activation
Okonkwo et al. [56]	122 (48⁺ ε4+)	40–65	Cross-sectional	Old/new recognition	ROI (precuneus, posterior cingulate)	(1) No difference in activation for familiarity effect (old > new)
Rajah et al. [68]	51 (11 ε4+)	41–58	Cross-sectional	Spatial context memory	Whole-brain	(1) Greater activation in right hippocampus during low-load encoding among ε4+ with positive FH (2) Greater encoding-related activation and lower retrieval related activation in fusiform cortex among ε4+ with positive FH
Trivedi et al. [78]	40 (23 ε4+)	40–65	Cross-sectional	Old/new recognition	Whole-brain & ROI (medial temporal lobe)	(1) Lower activation for novelty effect (new > old) in right hippocampus among ε4+ (2) No difference in activation for familiarity effect (old > new)
Xu et al. [79]	74 (33 ε4+)	40–65	Cross-sectional	Old/new recognition	Whole-brain	(1) Lower activation for familiarity effect (old > new) in left dorsal posterior cingulate/precuneus among ε4+ (2) Lower activation for faces (old or new) in left anterior cingulate gyrus among ε4+

*Note*. Sample *N* reflects the total number of cognitively healthy middle-aged participants reportedly included in relevant *APOE* ε4 analyses. The number of *APOE* ε4 carriers (ε4+) is provided in parentheses. Age range is reported in years and reflects the stated age range of the included participants or, if unavailable, the age range used for (initial) recruitment. We did not differentiate between studies adopting different definitions of *APOE* ε4+ (e.g., ε3/ε4 vs. ε3/ε4 + ε4/ε4). For studies with multiple groups (e.g., FH+/ε4+, FH-/ε4+, etc.), we summed across carrier/non-carrier groups. For studies reporting the percentage of *APOE* ε4 carriers, we used the reported sample sizes to calculate values and, where necessary, rounded to the nearest whole number (indicated by ⁺). Design reflects the cross-sectional or longitudinal nature of the *APOE* ε4 analysis and not necessarily the overall design of the study. For Task, we used our own labels and not necessarily those provided by the authors. This helps to highlight commonalities across studies, where they exist. Abbreviations: *APOE* = apolipoprotein-E; FH = family history of dementia; ROI = region of interest; RTs = reaction times.

Given the *APOE* ε4-AD link, it is unsurprising that most tasks were memory-related (*n* = 8, 72.7 %). Old/new recognition memory tasks were the most common (*n* = 5). Results from these studies were inconclusive and sometimes contradictory. For instance, while Johnson et al. [74] did not observe *APOE* ε4-related differences in novelty-related activation, Trivedi et al. [78] reported lower novelty-related activation in the right hippocampus among ε4 carriers relative to ε4 non-carriers. Further, when examining familiarity-related activation, Trivedi et al. [78] did not identify differences related to *APOE* ε4 (see also [56]), despite another study reporting lower activation among ε4 carriers (relative to ε4 non-carriers) in left dorsal posterior cingulate cortex/precuneus [79]. Finally, while Evans et al. [72] did not detect a significant *APOE* ε4 group difference, they did find that non-carriers – but not ε4 carriers – exhibited greater activation in left hippocampus at encoding for remembered vs. forgotten items. Caution is warranted, however, as this was the only study to examine activation during the encoding phase of an old/new recognition task.

Nonetheless, differences in encoding-related activation between those with and without ε4 have been reported in another midlife memory study, albeit using a different paradigm. Rajah et al. [68] used a spatial context memory task and scanned participants at encoding and retrieval. At encoding, participants viewed photographs of faces presented to the left or right of fixation and were required to remember the item and its location. At retrieval, participants were asked to indicate which face they previously saw on the left or right, depending on the cue. The number of items was manipulated, producing low- and high-load conditions. The authors reported greater activation in the right hippocampus during low-load encoding among ε4 carriers with a family history of AD relative to ε4 non-carriers with a family history of AD, as well as those without either risk factor. The authors also observed that, among participants with a family history of AD, possession of the ε4 allele was related to differential activation of fusiform cortex during encoding and retrieval.

In a separate study, Newton et al. [55] used a spatial memory task to explore the association between AD risk factors, including *APOE* ε4, and grid cell-like activation in posteromedial entorhinal cortex. Using ultra-high resolution (7T) MRI, the authors scanned participants from the PREVENT Dementia cohort [83] while they watched videos of themselves passively navigate through a three-dimensional room containing seven everyday items. Participants were instructed to learn the locations of these items, which changed across videos. The walls of the room, each decorated with items, did not change, providing orientation clues. At the end of each video, participants were presented with three images of each item – one in the correct location, two in incorrect locations – and asked to identify the correct one. For this review, the crucial finding was that *APOE* ε4 alone was not associated with grid cell-like activation in posteromedial entorhinal cortex. While this task differed substantially to that use by Rajah et al. [68], it is notable that only one of these studies identified an ε4 effect at midlife, despite both tasks requiring participants to identify the spatial location of previously seen items.

The final memory-related task focused on prospective memory: remembering to perform a future planned action [82]. In this study, participants were presented with playing cards, presented sequentially. They were required to press a button if the suit was hearts or spades (sort trials) but withhold their response if the suit was clubs or diamonds (withhold trials). While doing so, participants also had to remember to press a different button if the card was a seven regardless of the suit (prospective memory trial). The authors observed an association between *APOE* ε4 and activation evoked by this task but only on prospective memory trials. Specifically, during prospective memory trials, *APOE* ε4 carriers were found to exhibit lower levels of activation than non-carriers in extrastriate cortex and the right superior parietal lobe.

*APOE* ε4-related differences in extrastriate cortex function have also been reported using a non-mnemonic paradigm, potentially suggesting that the allele has a detrimental impact on aspects of the visual system at midlife. Evans et al. [67] used a covert attention task in which participants were asked to indicate whether an item appeared on the left or right of screen following a cue, which was predictive of item location (valid cue) or not (invalid cue). Evans et al. found that ε4 carriers demonstrated lower activation than non-carriers in extrastriate cortex independent of cue type (valid, invalid). However, in a subsequent study, the same group failed to observe differences in activation between groups when using the exact same covert attention task [82]. Accordingly, given the lack of internal replication, it is unclear whether differences in extrastriate cortex function are robust.

While nearly all tasks used to explore *APOE* ε4’s influence at midlife were memory- or attention-related, Johnson et al. [75] used a metacognitive self-appraisal task. In this paradigm, a series of adjectives were presented, and participants were asked to indicate whether the adjectives described their own traits/abilities (self-referential condition) or were of positive valence (non-referential condition). The authors did not identify *APOE* ε4-related differences, but their analysis was restricted to regions not showing an interaction between the allele and family history of AD. Despite not being the primary focus of this review, Johnson et al. did find that ε4 carriers without a family history of AD showed greater activation for referential self-appraisal in left superior frontal gyrus, left anterior cingulate, and retrosplenial areas in left superior frontal gyrus, left anterior cingulate gyrus, and retrosplenial cortex relative to all other groups. Yet, while differences between ε4 carriers/non-carriers without a family history of AD could point to an allele-specific impact at midlife, the lack of difference between ε4 carriers/non-carriers with a family history of AD complicates this interpretation.

### 3.5 Brain connectivity

Table 3 provides an overview of the seven midlife studies that examined differences in brain connectivity between middle-aged *APOE* ε4 carriers and non-carriers. Five reported significant ε4-related differences, representing a majority (71.4 %). However, the methods and, in turn, the form of connectivity probed varied across studies. One study used diffusion MRI [72], three studies used resting-state fMRI [71,73,76], while an additional three studies used some combination of the two [58,59,84].

**Table 3:** Summary of studies examining *APOE* ε4-related differences in brain structural/functional connectivity at midlife.

Study	Sample *N*	Age range	Design	Analytical approach	Key *APOE* ε4 result(s)
Dowell et al. [71]	37 (17 ε4+)	43–58	Cross-sectional	Whole-brain	(1) No difference in 8 resting-state networks
Evans et al. [72]	32 (16 ε4+)	45–55	Cross-sectional	ROI (hippocampus)	(1) No difference in hippocampal ODI
Fortel et al. [58]	76 (38 ε4+)	40–60	Cross-sectional	Whole-brain	(1) Global shift toward hyper-excitation in rs-SC among female (but not male) ε4+ (2) Lower tolerance to network dysfunction among female (but not male) ε4+
Fortel et al. [59]	76 (38 ε4+)	40–60	Cross-sectional	Whole-brain	(1) Global shift toward hyper-excitation in rs-SC among ε4+ (2) Shift toward hyper-excitation across several ROIs comprising the rs-SC among female (but not male) ε4+, starting at age 50
Goveas et al. [73]	46 (20 ε4+)	44–65	Cross-sectional	ROI (DMN: posterior cingulate, ECN: dorsolateral prefrontal cortex, SN: orbital anterior insula)	(1) Lower DMN connectivity among ε4+ (2) Lower ECN connectivity among ε4+ (3) Greater SN connectivity among ε4+
Korthauer et al. [84]	76 (38 ε4+)	40–60	Cross-sectional	Whole-brain	(1) Lower global and local efficiency in rs-SC among ε4+ (2) Greater betweenness centrality of right putamen and right thalamus, as well as greater participation coefficient of the right thalamus, in rs-SC among ε4+ (3) Lower betweenness centrality of left lateral orbitofrontal cortex and left superior temporal gyrus, as well as lower participation coefficient of the left lateral orbitofrontal cortex and left superior frontal cortex, in rs-SC among ε4+ (4) In targeted failure analysis, greater resilience evident in ε4+ until 10 % of hubs removed, then lower resilience as central nodes removed
Li et al. [76]	46 (20 ε4+)	44–65	Cross-sectional	ROI (hippocampus)	(1) Lower functional connectivity between hippocampus and various regions (caudate, thalamus, posterior cingulate, superior frontal gyrus, lentiform nucleus, medial frontal gyrus, insula, anterior cingulate corte, culmen of cerebellum) among ε4+

*Note*. Sample *N* reflects the total number of cognitively healthy middle-aged participants reportedly included in relevant *APOE* ε4 analyses. The number of *APOE* ε4 carriers (ε4+) is provided in parentheses. Age range is reported in years and reflects the stated age range of the included participants or, if unavailable, the age range used for (initial) recruitment. We did not differentiate between studies adopting different definitions of *APOE* ε4+ (e.g., ε3/ε4 vs. ε3/ε4 + ε4/ε4). For studies with multiple groups (e.g., FH+/ε4+, FH-/ε4+, etc.), we summed across carrier/non-carrier groups. For studies reporting the percentage of *APOE* ε4 carriers, we used the reported sample sizes to calculate values and, where necessary, rounded to the nearest whole number (indicated by ⁺). Design reflects the cross-sectional or longitudinal nature of the *APOE* ε4 analysis and not necessarily the overall design of the study.

Abbreviations: *APOE* = apolipoprotein-E; DMN = default mode network; ECN = executive control network; FH = family history of dementia; ODI = orientation dispersion index; ROI = region of interest; rs-SC = resting-state structural connectome; SN = salience network.

The study to exclusively use diffusion MRI did so to examine *APOE* ε4-related differences in bilateral hippocampal microstructure, assessed via the orientation dispersion index (ODI; [72]). However, the authors found that ε4 carriers and non-carriers did not differ in bilateral hippocampal ODI. Nevertheless, Evans et al. did observe a difference between groups in the association between hippocampal ODI and old/new recognition memory, with a positive trend evident only for ε4 non-carriers. It is possible, therefore, that *APOE* ε4 may not impact the microstructural properties of the hippocampus but may impact how these properties relate to and support memory.

Among the three studies to exclusively use resting-state fMRI, there was variation in the analytical methods used. Goveas et al. [73] and Li et al. [76] both conducted seed-based analyses, examining differences in functional connectivity associated with specific regions. Goveas et al. focused on core components of different resting-state functional networks: posterior cingulate cortex (default mode network), dorsolateral prefrontal cortex (executive control network), and orbital anterior insula (salience network). Li et al., conversely, focused on the hippocampus. Despite this, both studies reported patterns of lower functional connectivity among ε4 carriers relative to non-carriers; Goveas et al. identified this pattern within the default mode and executive control networks, while Li et al. identified this pattern between the hippocampus and several regions. The exception was that of the salience network, where Goveas et al. identified increased connectivity among ε4 carriers relative to non-carriers. By contrast, Dowell et al. [71] used independent components analysis to identify resting-state networks and test for *APOE* ε4-related differences. Although the authors identified eight such networks, including the default mode and executive control networks, no carrier/non-carrier differences were evident in their midlife sample. This contradicts the results reported by Goveas et al., raising questions about the role of methodological choices.

Three studies have adopted an alternative approach, combining both diffusion MRI and resting-state fMRI [58,59,84]. For example, Korthauer et al. [84] extracted graph theoretical properties from three networks – a functional network derived from resting-state fMRI, a structural network derived from diffusion MRI, and an integrated functional-structural network (the “resting-state structural connectome”) – and then examined differences between middle-aged *APOE* ε4 carriers and non-carriers. While the authors did not find differences in the properties of the functional and structural networks, they observed differences in the resting-state structural connectome. Specifically, they found that ε4 carriers exhibited lower levels of global and local efficiency in this combined network. Korthauer et al. also reported differences in the properties of hub networks and lower resilience to node failure, especially once more than 10 % of hubs were removed. These results highlight the potential utility of combining resting-state fMRI and diffusion MRI to probe connectivity and indicate that such an approach may reveal subtle *APOE* ε4-related differences at midlife. Concordantly, two further studies have demonstrated that *APOE* ε4 carriers, in particular female *APOE* ε4 carriers, exhibit a global shift towards hyper-excitation in the resting-state structural connectome [59] and a lower tolerance to network dysfunction [58] at midlife.

## 4 Discussion

### 4.1 Summary of findings

In this scoping review, we synthesized research examining *APOE* ε4-related differences in brain structure, function, and connectivity at midlife. The reviewed MRI studies provide little evidence to indicate that middle-aged *APOE* ε4 carriers exhibit consistent differences in brain structure relative to non-carriers. Variation in study design, analytical approach, and outcome measure do not appear to offer a coherent explanation for these null findings. Among studies assessing brain function and connectivity, *APOE* ε4-related differences were more commonly reported. However, there was marked variation in the regions/networks implicated and the direction of the reported differences. The neural profile of *APOE* ε4 at midlife thus remains poorly characterized.

The absence of robust, consistent differences in the reviewed studies may reflect a genuine lack of *APOE* ε4-related differences in the brain at midlife. Such an explanation is consistent with the prodromal hypothesis, which proposes that ε4-related differences in the brain and cognition emerge relatively late in the adult lifespan, driven by the heightened burden of AD pathology among ε4 carriers who will go on to develop the disease [24,25]. In this light, it is perhaps unsurprising that the reviewed studies reported many null findings. However, the lack of a distinct neural profile among middle-aged *APOE* ε4 carriers is also arguably consistent with the antagonistic pleiotropy hypothesis [39,40]. Although some *APOE* studies have claimed to identify examples of antagonistic pleiotropy in midlife (e.g., [41]), the hypothesis – as originally stated – predicts subtle differences, if any, at midlife, as the allele’s influence shifts from advantageous to disadvantageous [42], [85]. It follows, therefore, that the reviewed studies do not necessarily provide evidence that *APOE* ε4-related differences are driven entirely by pathology. Longitudinal studies are needed to determine whether midlife differences in MRI-based measures, if present, are related to prodromal AD, a necessary step for the development of imaging biomarkers [26].

It is less clear how lifespan accounts can explain the results from the reviewed studies. These accounts propose that the *APOE* ε4 allele induces a prolonged pattern of hyperactivation that is present across early- and mid-life, predisposing vulnerable brain networks to AD pathology and later dysfunction [34,35]. Yet, consistent with a review of studies on older adults [23], the reported differences in patterns of task-evoked fMRI activation were extremely mixed. In fact, of the 10 fMRI studies identified, only two observed greater activation in middle-aged *APOE* ε4 carriers compared to non-carriers [68], [75]. Four studies even reported the opposite pattern, linking *APOE* ε4 with lower levels of task-evoked activation [67], [78], [79], [82]. While the use of different paradigms and stimuli place constraints on comparability across studies, it is nevertheless challenging to reconcile these findings with claims that fMRI hyperactivation observed in young, at-risk adults (e.g., [29]) is maintained into midlife.

Another possibility is that the ε4 allele impacts brain health at midlife through alternative mechanisms. For example, *APOE* ε4 has been associated with cerebrovascular dysfunction in aging and AD, including alterations in cerebral blood flow and blood-brain barrier integrity [36], [86]. Differences between ε4 carriers and non-carriers in these vascular mechanisms have likewise been observed in studies of young and middle-aged adults [87], [88], [89], [90]. Such findings suggest that *APOE* ε4’s influence on the cerebrovascular system is more prominent than its influence on brain structure, function, or connectivity. Consistent with this notion, one large cross-sectional study of late middle-aged and older adults reported that *APOE* ε4 was associated with greater white matter hyperintensity volumes, an indicator of poorer cerebrovascular health, but not gray matter volume or white matter microstructure [16]. Together, these findings provide evidence that *APOE* ε4 is linked to midlife brain health but, crucially, not in the neural properties targeted in this scoping review.

A variety of methodological issues could have influenced the results reported, however, contributing to a lack of ε4-related differences in brain structure and/or variability in ε4-related differences in brain function/connectivity. One prominent issue is the widespread use of small samples, which limits statistical power and increases the likelihood of missing a true “effect” (i.e., a Type II error) [91]. In the reviewed studies, the median total sample size was 92.5, which is comparable to *APOE* ε4-related studies in young adults (*Mdn* = 62; [92]) but provides insufficient power to reliably detect small effects. This is relevant here as effect sizes associated with common genetic variants on neuroimaging outcomes are typically small [93]. While *APOE* ε4 may represent an exception to this rule, a recent study of ∼43,000 late middle-aged and older adults casts some doubt on this [21]. It is possible, therefore, that *APOE* ε4 does influence brain structure, function, and/or connectivity at midlife, but most studies were simply underpowered to detect it.

Another methodological issue was the lack of consideration for the roles of sex and ethnocultural diversity. Regarding sex, we found that most studies included more females than males, some to a greater extent that others, but few actively explored the influence of sex or sex-related factors. Indeed, only four studies identified in this review even mentioned menopause, despite the focus on midlife. This was surprising as the *APOE* ε4 allele has long been shown to have a greater impact on AD risk among females [8], especially among those aged 60–75 years [94,95], potentially underpinned by estrogen-*APOE* interactions [96,97]. Regarding ethnocultural diversity, we found that few studies reported the racial/ethnic breakdown of their samples. Among those that did, there was a clear skew towards white or “Caucasian” individuals. Although unsurprising given the broader cognitive neuroscience literature, the lack of studies including non-white individuals limits the generalizability of results [98]. This is particularly problematic for research on *APOE*, as evidence suggests that the association between the ε4 allele and AD risk varies as a function of race/ethnicity [7], [8], [94]. Given this, we cannot rule out the possibility that inconsistencies across studies were partly related to the differential composition of the participants, both in terms of sex and ethnocultural diversity, as well as other health factors (e.g., hormone replacement therapy, body mass index, hypertension). Future research would greatly benefit from the inclusion of more diverse midlife samples, as well as a greater consideration of menopause and related factors.

### 4.2 Limitations

Our scoping review has some limitations that should be considered. First, we focused exclusively on studies published in peer-reviewed journals. It is thus possible that we missed relevant research, which may be available as pre-prints, conference proceedings, or dissertations. If publication bias was present, our focus on peer-reviewed studies may have resulted in the over-representation of work reporting significant *APOE* ε4-related differences. However, the studies reviewed – many reporting null findings – cast some doubt on this. Second, our scoping review included studies published as of July 11, 2024. Although this is an unavoidable feature of reviews, we may have missed relevant work published since this date. Third, we included studies only if the age range was reported and, crucially, if it fell between 40–65 years, a common definition of midlife [43]. While we identified 30 studies meeting this criterion, this led to the exclusion of several studies that claimed to focus on midlife. In this regard, our approach arguably placed a greater emphasis on specificity than sensitivity (although see Supplementary Material).

## 5 Conclusions

The *APOE* ε4 allele is a major genetic risk factor for late-onset Alzheimer’s disease, yet its influence on the middle-aged brain is not well characterized. In this scoping review, we aimed to address this by synthesizing research examining ε4-related differences on MRI-derived measures of brain structure, function, and connectivity among middle-aged adults. While the current literature is dominated by small, non-representative samples, the available evidence suggests that *APOE* ε4 carriers and non-carriers do not exhibit robust, consistent differences at midlife. This is especially true for measures of brain structure, although the allele’s influence on brain function and connectivity remains poorly delineated.

## Funding

This work was supported by Canada Research Chairs Program CRC-2022–00,240, CIHR Sex & Gender Research Chair GS9–171,369, and NSERC Discovery Grant RGPIN-2018–05,761 awarded to M. N. Rajah, and a Canadian Consortium on Neurodegeneration in Aging (CCNA) – Women, Sex, Gender, & Dementia (WSGD) Postdoctoral Fellowship awarded to R. Lissaman.

## Declaration of generative AI and AI-assisted technologies in the writing process

During the preparation of this work, the first author (R. Lissaman) used ChatGPT to rephrase limited sections of text, with the aim of improving manuscript readability. After using this tool/service, the author reviewed and edited the content as needed and takes full responsibility for the content of the published article.

## CRediT authorship contribution statement

**Rikki Lissaman:** Writing – review & editing, Writing – original draft, Visualization, Project administration, Methodology, Investigation, Formal analysis, Data curation, Conceptualization. **Sidra Anjum:** Writing – review & editing, Investigation, Formal analysis. **Andrea Quaiattini:** Resources, Methodology. **M. Natasha Rajah:** Writing – review & editing, Writing – original draft, Supervision, Methodology, Conceptualization.

## Declaration of competing interest

The authors declare that they have no known competing financial interests or personal relationships that could have appeared to influence the work reported in this paper.

## Appendix Supplementary materials

Image, application 1

## Footnotes

1 The terms “sex” and “gender” were used somewhat interchangeable in the reviewed studies. For consistency, we use the term sex throughout this scoping review. Further, as the included studies overwhelmingly used self-report to categorize individuals as male/female or man/woman, we also use terms such as “mixed-sex studies” and “single-sex studies”. This reflects the state of the literature, which has hitherto largely ignored intersex individuals, as well as transgender and gender non-binary individuals.
