# Supplementary material for: APOE ε4-related differences in brain structure, function, and connectivity at midlife: A scoping review
Source: J Prev Alzheimers Dis. 2025 Aug 29;12(9):100364. doi: 10.1016/j.tjpad.2025.100364 (PMC12501331; doi:10.1016/j.tjpad.2025.100364)
Supplement: Supplementary file 1 [file mmc1.docx]

**SUPPLEMENTARY MATERIAL**

***APOE* ε4-related differences in brain structure, function, and connectivity at midlife:**

**A scoping review**

Rikki Lissaman^a,b,*^, Sidra Anjum^c^, Andrea Quaiattini^d^, and M. Natasha Rajah^a,e^

^a^Department of Psychiatry, McGill University, Montreal, QC, Canada

^b^Department of Psychology, Royal Holloway, University of London, Egham, Surrey, UK

^c^Centre for Addiction and Mental Health, Toronto, ON, Canada

^d^Schulich Library of Physical Sciences, Life Sciences, and Engineering, McGill University, QC, Canada

^e^Department of Psychology, Toronto Metropolitan University, Toronto, ON, Canada

*Corresponding author: Rikki Lissaman ([rikki.lissaman@mail.mcgill.ca](mailto:rikki.lissaman@mail.mcgill.ca))

**Table of Contents**

[Search Strategy 3](#_Toc206428757)

[Ovid MEDLINE(R) ALL <1946 to July 10, 2024> 3](#_Toc206428758)

[Embase Classic+Embase <1947 to 2024 July 10> 4](#_Toc206428759)

[APA PsycInfo <1967 to July Week 1 2024> 5](#_Toc206428760)

[Scopus 6](#_Toc206428761)

[Age Range Analysis 7](#_Toc206428762)

[Figures 9](#_Toc206428763)

[Figure 1 9](#_Toc206428764)

# **Search Strategy**

## **Ovid MEDLINE(R) ALL <1946 to July 10, 2024>**

1. apolipoprotein e/ or apolipoprotein e2/ or apolipoprotein e3/ or apolipoprotein e4/ [6874]
2. (apolipoprotein*-e* or apoe* or apo-e* or apoprotein*-e*).ti,ab,kf [39993]
3. 1 or 2 [40612]
4. functional neuroimaging/ or brain mapping/ or connectome/ or magnetic resonance imaging/ or diffusion magnetic resonance imaging/ or diffusion tensor imaging/ [576203]
5. ((magnetic resonance adj3 (imag* or scan or scans or scanning)) or ((diffusion weight* or diffusion tensor) adj3 (imag* or scan* or scans or scanning)) or ((mr or nmr) adj (imag* or scan or scans or scanning)) or (((funct* or intrinsic) adj1 connect*) or mri* or fmri* or f-mri* or nmri or dmri* or d-mri* or dti or dwi or rsfmri* or resting-state)).ti,ab,kf [632225]
6. 4 or 5 [841108]
7. 3 and 6 [2936]
8. 7 not (exp animals/ not humans.sh.) [2717]

## **Embase Classic+Embase <1947 to 2024 July 10>**

1. apolipoprotein e/ or apolipoprotein e2/ or apolipoprotein e3/ or apolipoprotein e4/ [47664]
2. (apolipoprotein*-e* or apoe* or apo-e* or apoprotein*-e*).ti,ab,kf [56667]
3. 1 or 2 [68509]
4. functional neuroimaging/ or brain mapping/ or connectome/ or nuclear magnetic resonance imaging/ or diffusion weighted imaging/ or diffusion tensor imaging/ [1164151]
5. ((magnetic resonance adj3 (imag* or scan or scans or scanning)) or ((diffusion weight* or diffusion tensor) adj3 (imag* or scan* or scans or scanning)) or ((mr or nmr) adj (imag* or scan or scans or scanning)) or (((funct* or intrinsic) adj1 connect*) or mri* or fmri* or f-mri* or nmri or dmri* or d-mri* or dti or dwi or rsfmri* or resting-state)).ti,ab,kf [962972]
6. 4 or 5 [1403377]
7. 3 and 6 [6719]
8. 7 not ((exp animal/ or animal experiment/ or nonhuman/) not (exp human/ or human experiment/)) [6199]

## **APA PsycInfo <1967 to July Week 1 2024>**

1. Apolipoprotein E/ [3180]
2. (apolipoprotein*-e* or apoe* or apo-e* or apoprotein*-e*).ab,id,ti [7910]
3. 1 or 2 [7943]
4. Brain Connectivity/ or magnetic resonance imaging/ or diffusion tensor imaging/ or functional magnetic resonance imaging/ [66106]
5. ((magnetic resonance adj3 (imag* or scan or scans or scanning)) or ((diffusion weight* or diffusion tensor) adj3 (imag* or scan* or scans or scanning)) or ((mr or nmr) adj (imag* or scan or scans or scanning)) or (((funct* or intrinsic) adj1 connect*) or mri* or fmri* or f-mri* or nmri or dmri* or d-mri* or dti or dwi or rsfmri* or resting-state)).ab,id,ti [114487]
6. 4 or 5 [118226]
7. 3 and 6 [1312]

## **Scopus**

( TITLE-ABS ( "apolipoprotein*-e*" OR "apoe*" OR "apo-e*" OR "apoprotein*-e" ) ) AND ( ( TITLE-ABS ( ( ( "magnetic resonance" ) W/3 ( imag* OR scan OR scans OR scanning ) ) ) ) OR ( TITLE-ABS ( ( ( "diffusion weight*" OR "diffusion tensor" ) W/3 ( imag* OR scan* OR scans OR scanning ) ) ) ) OR ( TITLE-ABS ( ( ( mr OR nmr ) PRE/1 ( imag* OR scan OR scans OR scanning ) ) ) ) OR ( TITLE-ABS ( ( ( funct* OR intrinsic ) W/1 connect* ) ) ) OR ( TITLE-ABS ( mri* OR fmri* OR "f-mri*" OR nmri OR dmri* OR "d-mri*" OR dti OR dwi OR rsfmri* OR "resting-state" ) ) )

# **Age Range Analysis**

During full-text screening, we excluded 62 studies for failing to report an age range. The logic behind this exclusion, outlined in our pre-registered protocol, was straightforward: means and standard deviations (or standard errors^[[1]](#footnote-1)^) do not prove conclusively that individuals younger than 40 or older than 65 were excluded from the to-be-reviewed studies. Thus, by requiring that studies report an age range, we were able to ensure that our review focused on midlife-specific differences between *APOE* ε4 carriers and non-carriers. However, we acknowledge that our approach placed a greater emphasis on specificity than sensitivity and could, in some cases, lead to relevant studies being excluded for simple differences in reporting standards. As such, we re-assessed each of the 62 studies. Specifically, for each study, we extracted the groups means and standard deviations, estimated an age range using these values (*M* ± 2 SDs), and then compared this against our pre-stated cut-offs (40-65 years). This approach relies on the assumption that age is normally distributed, something that is often difficulty – if not impossible – to evaluate from the available data. Nevertheless, this approach does provide some insight as to the suitability of our approach.

Of the 62 studies examined, we found that 36 (58.1%) reported group means that were outside our pre-stated range (e.g., mean age = 75.1). A further 23 studies (37.1%) reported group means that fell within 40-65 years but, when accounting for the reported standard deviations, likely included participants below 40 or above 65 years. This left 3 studies (4.8%) whose estimated age ranges were acceptable. However, two of these studies compared “high” and “low” AD risk groups, which was defined by family history or a combination of family history and *APOE* ε4 (i.e., there was no *APOE* ε4 comparison). These studies, therefore, were not relevant for this scoping review. The one possible exception was Brenowitz et al. (2023). Technically, this study did not report the relevant figures (*M*s, *SD*s) for *APOE* ε4 carriers and non-carriers, nor for their overall MRI sample (i.e., the most relevant sample for this review). However, the full sample had a mean age of 55.5 and a standard deviation of 3.3, which is consistent with a range within 40-65 years (assuming a normal distribution for age). It is notable that this excluded study – the only one arguably consistent with our definition of midlife – also found no association between *APOE* ε4 and brain health outcomes, namely total brain volume and hippocampal volume.

In sum, this supplementary analysis suggests that our pre-registered age criterion did not lead the exclusion of significant midlife-specific studies.

**References**

Brenowitz, W. D., Fornage, M., Launer, L. J., Habes, M., Davatzikos, C., & Yaffe, K. (2023). Alzheimer’s disease genetic risk, cognition, and brain aging in midlife. *Annals of Neurology*, *93*(3), 629–634. <https://doi.org/10.1002/ana.26569>

# **Figures**

## **Figure 1**

*Total Sample Size as a Function of Publication Year.*

#
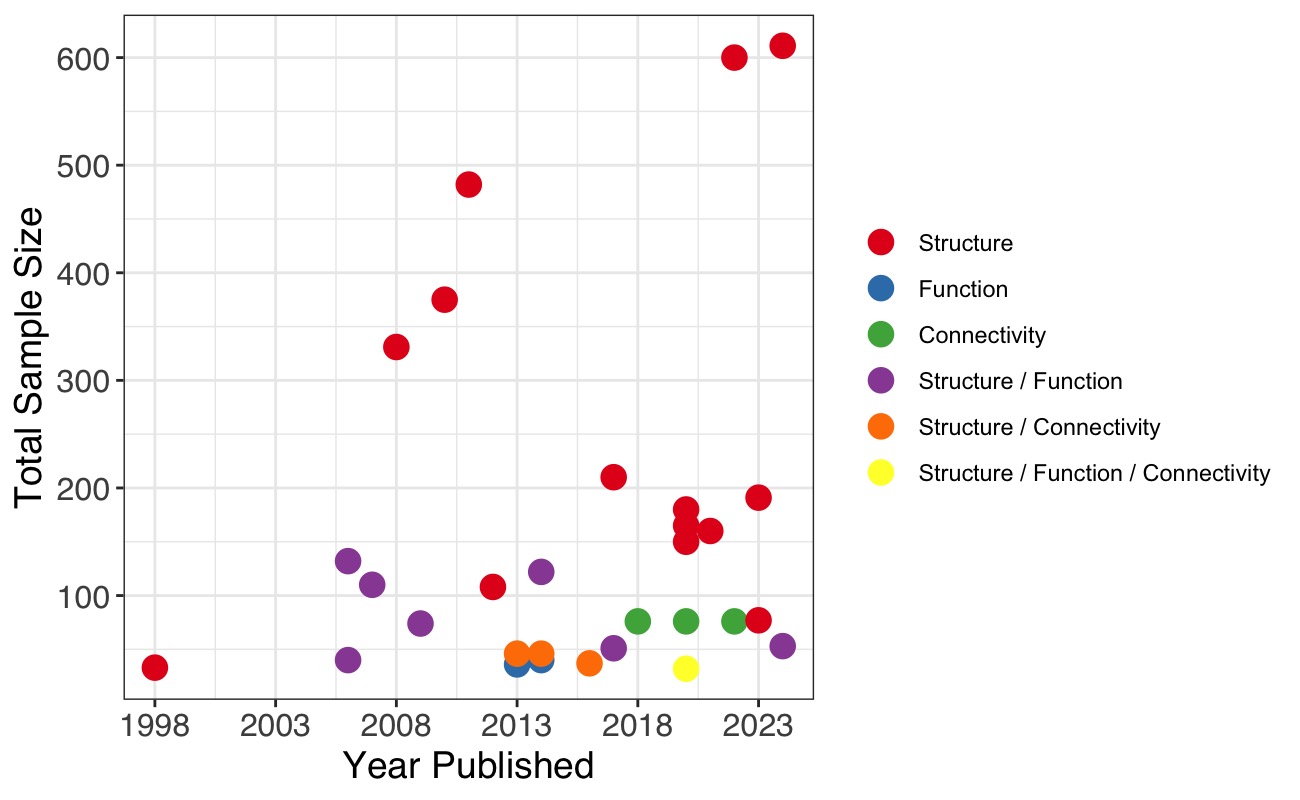


*Note*. Total sample size and publication year for midlife studies examining differences between *APOE* ε4 carriers and non-carriers. Each circle represents an individual study and is colored according to the neural properties examined.

1. If standard errors were reported for age, we multiplied the reported values by the square root of the sample size to obtain standard deviations (SD = SE x √*n*). [↑](#footnote-ref-1)
